# Congenital generalized lipodystrophy in two siblings from Saudi Arabia: A case report

**DOI:** 10.1002/ccr3.5720

**Published:** 2022-04-20

**Authors:** Abdulrrahman Hummadi, Ahmed Ali Nahari, Ali Jaber Alhagawy, Ibrahim Zakri, Raed Abutaleb, Saeed Yafei

**Affiliations:** ^1^ Jazan Endocrinology and Diabetes Center Ministry of Health Jazan Saudi Arabia; ^2^ King Fahd Diabetes and Endocrinology Center King Fahd hospital Jazan Saudi Arabia; ^3^ Jazan Health Affairs Ministry of Health Jazan Saudi Arabia; ^4^ Faculty of Medicine and Health Sciences Internal Medicine Department Taiz University Taiz Yemen

**Keywords:** AGPAT2, Berardinelli–Seip congenital lipodystrophy, lipodystrophy

## Abstract

Congenital generalized lipodystrophy type 1 (CGL1) is a very rare autosomal recessive genetic mutation with generalized lipoatrophy and metabolic complications. We report CGL1 in two Saudi female siblings with lipoatrophy, diabetes mellitus, hypertriglyceridemia, steatohepatitis, and acanthosis due to very rare homozygous 1‐acylglycerol‐3‐phosphate O‐acyltransferase β (AGPAT2) genetic variant.

## 1 INTRODUCTION

Lipodystrophy syndromes are rare heterogeneous disorders, inherited or acquired, and characterized by partial or generalized loss of adipose tissue with subsequent development of insulin resistance and its complications. Genetic lipodystrophies are inherited in autosomal recessive or autosomal dominant pattern.^1^, ^2^ The two most common subtypes of genetic lipodystrophies are congenital generalized lipodystrophy (CGL) and familial partial lipodystrophy (FPLD).^3^

CGLs, or Berardinelli–Seip congenital lipodystrophy (BSCL), are autosomal recessive disorders frequently recognized at birth or shortly thereafter.^4^ At least four molecularly different genetic mutations of congenital lipodystrophy have been defined, including AGPAT2, BSCL2, CAV1, and polymerase I and transcript release factor (PTRF). Mutations of 1‐acylglycerol‐3‐phosphate *O*‐acyltransferase 2 (AGPAT2) in CGL type 1 and seipin in CGL type 2 are responsible for 95 percent of reported cases.^5^, ^6^ The severity of these metabolic derangements is mostly proportional to the extent of body fat loss.^4^

People with CGL usually have generalized lipoatrophy and metabolic complications related to insulin resistance and abnormal deposition of fat in other organs such as the liver, skeletal muscles, and the heart. Complications may include diabetes mellitus, hypertriglyceridemia, steatohepatitis, acanthosis nigricans, and hypertension.^7^ Other features include accelerated growth in childhood, acromegaloid appearance, hepatosplenomegaly, umbilical hernia, and, in women, polycystic ovary syndrome, clitoromegaly, and hirsutism.^8^ CGL patients usually have extremely low serum leptin levels with increased basal metabolic rate and voracious appetite.^7^

The prevalence of CGL in Saudi Arabia is unknown; only four cases were reported in the literature, although the true prevalence may be higher due to the high prevalence of consanguinity in this country.^9^, ^10^, ^11^ Worldwide, there are only hundreds of reported cases of CGL in the literature and the estimated prevalence of lipodystrophy is around 1.3–4.7 cases/million.^12^ We herein reported the clinical and molecular characteristics of two Saudi female siblings with CGL type 1 due to frameshift homozygous AGPAT2 variant c.158del/p.(Gly53Alafs*8) identified in AGPAT2 gene.

## 2 CASE PRESENTATION

This study was reviewed and approved by the institutional ethical committee, Jazan, Saudi Arabia, and was conducted in agreement with the Declaration of Helsinki Principles. The two sisters are offspring of a consanguineous Saudi family living in Jazan. The written informed consent was obtained from patients and their father.

Two sisters, 22 and 14 years old, were referred to diabetes and endocrinology center in Jazan, Saudi Arabia, with history of uncontrolled diabetes mellitus, HbA1c (12% and 14.1%, respectively), hypertriglyceridemia, and hepatomegaly. Both were on high‐dose insulin, total doses of 200 iu/day in the older and 290 iu/day in the younger girl. Diabetes had been diagnosed at the age of 13 and 8 years, respectively. They had never achieved good glycemic control despite high‐dose insulin and oral antidiabetic medications. Their history did not reveal ketoacidosis despite continuous high blood sugar. The older sister had a history of admission to a regional hospital due to an episode of acute pancreatitis. Family history revealed a consanguineous relationship between their parents who did not complain of any chronic medical disease. The older sister menarche started at the age of 12, and then, she had irregular periods (oligomenorrhea) with scanty menstruation; the younger sister had not yet started her menstrual periods. Ovarian ultrasound reveals appearance of multiple small cysts, so these in addition to oligomenorrhea and androgenic features signify that she has polycystic ovary. Hepatic fibroscan and DEXA scanning were not performed as they are not available in our center.

On physical examination, the older sister height was 160 cm, weight 46.8 Kg, and body mass index (BMI) 18 kg/m^2^ and the younger height was 154 cm, weight 41 Kg, and BMI at the 24^th^ percentile for age and sex. Systolic and diastolic arterial blood pressures were in the normal range for their age, and they are not on antihypertensive medications.

Both had clear patterns of generalized fat loss on cheeks, trunks, upper and lower extremities, acromegaloid features, hypertrophy of the skeletal muscles, prominent subcutaneous veins, acanthosis nigricans, and hirsutism. Their livers were palpable more than 3 cm below the right costal margins.

Laboratory tests presented in Table 1 revealed high levels of fasting serum glucose and HbA1c, and triglyceride levels with mild evaluation of liver enzymes. Investigations also included fasting c‐peptide, insulin levels, thyroid‐stimulating hormone, glutamic acid decarboxylase antibody, fasting leptin, and adiponectin levels. An abdominal ultrasound scan showed an enlarged liver in both. However, further cardiac evaluation with echocardiography revealed no cardiac involvement. X‐ray of the upper and lower limbs did not reveal bone cystic lesions in both sisters.

**TABLE 1:** Anthropometric and biochemistry data of the two cases

Variables	Case 1	Case 2	Reference range
Age, years	22	14	
BMI	18	24^th^ percentile	
Systolic blood pressure	132	135	
Diastolic blood pressure	85	80	
Diabetes duration, years	9	5	
Fasting plasma glucose, mg/dl	402	391	70–99
HbA1c, %	12	14.1	<5.7
C‐peptide, ng/mL	2.6	2.3	1.1–4.4
Insulin, mIU/L	27	31	0–25
Anti‐GAD antibodies, U/ml	<5	<5	<10
Total cholesterol, mg/dl	185	286	<200
HDL cholesterol, mg/dl	24	16	>50
LDL cholesterol, mg/dl	56	83	<130
Triglycerides, mg/dL	503	574	<150
Leptin, ng/mL	1.2	1.8	3.7–11.1
Adiponectin, ng/mL	2	2.1	8.2–19
Creatinine kinase, U/L	26	25	29–168
ALT, U/L	95	108	<40
AST, U/L	66	59	<40
GGT, U /L,	130	152	<38
Creatinine, mg/dl	0.3	0.3	0.6–1.2
Urea, mg/dl	7	9	15–45
Uric acid, mg/dl	4.2	5.3	2.7–7.3
Urine ACR, mcg/mg	23	12	0–30

Abbreviations: ACR, albumin creatinine ratio; ALT, alanine aminotransferase; AST, aspartate aminotransferase; BMI, body mass index; GAD, glutamic acid decarboxylase; GGT, gamma‐glutamyl transpeptidase; HbA1c, hemoglobin A1c; HDL, high‐density lipoprotein; and LDL, low‐density lipoprotein.

A clinical diagnosis of CGL was suspected, and genetic analysis was performed, which confirmed autosomal recessive GCL type 1 with a homozygous variant identified at AGPAT2 gene, c.158del/p.(Gly53Alafs*8) in both sisters. This genetic variant can predict the change of the amino acid glycine residue at position 53 with alanine creating a shift in the reading frame starting at codon 53 and ending in a stop codon 7 positions downstream.

Current management includes nutritional and exercise program in addition to metformin, multiple daily insulin injections, statin, and fenofibrates. They are scheduled for close follow‐up aiming to control metabolic comorbidities and prevent micro‐ and macrovascular complications and pancreatitis.

## 3 DISCUSSION

AGPATs are key enzymes required for triglyceride and phospholipid biosynthesis, and for the growth and development of adipocytes.^13^ AGPAT2 is an enzyme located on chromosome 9q34 and highly expressed in adipose tissue, and its insufficiency may cause lipodystrophy by restricting triglyceride or phospholipid biosynthesis within the tissue.^2^

Patients with CGL type 1 are either homozygous or compound heterozygous for AGPAT2 gene mutations and inherited in autosomal recessive pattern. Heterozygous carriers are asymptomatic and are at increased risk of diabetes.^14^ Most cases of AGPAT2 mutations are reported from consanguineous families in Brazil, Lebanon, and Scandinavia.^3^, ^12^ Most of the AGPAT2 mutations include frameshifts or altered mRNA splicing producing non‐functional enzyme.^15^

Our two cases have pathogenic homozygous variants recognized at AGPAT2 gene, c.158del/p.(Gly53Alafs*8). This genetic variant was reported in one Emirati female presented with diabetes firstly diagnosed at 14 years of age with hypertriglyceridemia and acanthosis.^16^ Another AGPAT2 homozygous variant, c.335del/p.(pro112argfs*39), was detected in two unrelated CGL type 1 patients from Saudi Arabia.^9^

Adipose tissue can be divided into mechanical and metabolic types.^17^ Mechanical adipose tissue supports and protects regions subjected to mechanical insults and includes fats in palms, soles, orbits, and periarticular area. Metabolic fat plays a role in the storage and release of energy and includes subcutaneous, intermuscular, intra‐abdominal, and intrathoracic fat in the bone marrow. Patients with CGL type 1 lack metabolically active fat, but mechanical fat is well preserved, whereas patients with CGL type 2 usually are deficient in both mechanical and metabolic adipose tissues.^5^

The clinical presentation of CGLs could be very heterogeneous with variation in the correlation between genotype and phenotype of these syndromes.^8^ The deficiency of metabolic adipose tissue induces ectopic deposition of fat in liver, skeletal muscles, and other insulin target tissues causing hepatomegaly, muscle hypertrophy, and insulin resistance.^7^, ^18^ That generalized fat loss on face, trunks, and upper and lower extremities with hypertrophy of the skeletal muscles in the limbs was clear in both sisters and started gradually in their first years of life, but these dysmorphic features did not attract clinical attention except after the development of diabetes. Type 1 CGL patients usually have average mental functions and gradual development of metabolic complications, in opposite to type 2 CGL who have mental retardation, and prominent dysmorphic and metabolic complications from the neonatal period^6^; this also could explain the delay in the diagnosis of such cases.

The gradual progress of ectopic fat deposition, hyperinsulinemia, and insulin resistance increased in the second decade of life making the diabetes difficult to control with conventional therapy. Our patients were diagnosed with diabetes at 8 and 13 years of age and were uncontrolled despite oral therapy and high insulin doses. The very high triglyceride level could be explained by insulin resistance and defective AGPAT2 function.^4^ Acromegaloid features are related to the growth‐promoting effects of extreme hyperinsulinemia due to direct effect on insulin receptors and indirect effect on insulin‐like growth factor I receptors.^1^

Hepatic involvement in these two siblings manifested with mild elevations of liver enzymes and hepatomegaly attributed to the accumulation of triglycerides in the livers. Another mechanism of steatohepatitis hypothesized that functional insufficiency of AGPAT2 could induce upregulation of liver activity of AGPAT1 enzyme isoform leading to overproduction of triglycerides with subsequent steatosis and hepatomegaly.^15^

Adipocytes secrete several bioactive substances including leptin, adiponectin, and tumor necrosis factor‐α, which play an important role in the pathophysiology of insulin resistance and dyslipidemia.^17^ The very low level of leptin and adiponectin in our patients is consistent with previous reports.^7^, ^15^ Serum leptin levels reflect the amount of adipose tissue in the body and correlate positively with adiposity. The low adiponectin level is linked negatively to insulin resistance.^19^

Management in these patients includes lifestyle modifications, diet regulation, control of metabolic complications, and early screening for other comorbidities. Insulin is used combined with metformin, and continuous glucose monitoring is performed aiming to control metabolic comorbidities. Although not prescribed for our patients due to high cost and unavailability in the health system, recombinant leptin is the most promising treatment option for patients with CGL. Metreleptin decreases hyperphagia with marked improvement of glucose levels, triglycerides, and steatohepatitis^6^; we hope it will be available for them in the near future.

## 4 CONCLUSION

In summary, we report two sisters with CGL type 1 due to AGPAT2 variant c.158del/p.(Gly53Alafs*8) frameshift mutation. The description of the clinical, biochemical, and molecular features of these cases is of great importance for the clinicians to build up data regarding this disease and for early diagnosis and appropriate screening and prevention of complications.

## CONFLICT OF INTEREST

The authors declare that they have no competing interests.

## AUTHOR CONTRIBUTIONS

AH planned the study. AAN supervised the molecular testing. AGH contributed to data interpretation and clinical investigations. RAT provided the clinical care and counseled the family. IZ critically reviewed the manuscript. SY wrote the draft of the manuscript and coordinated the research plan. All authors agreed on the final manuscript.

## CONSENT

The written informed consent was obtained from the patient and their father for publication of this case report.

## Data Availability

All the necessary information is provided within the manuscript.
